# Global science meets ethnic diversity: Ian McGonigle interviews GenomeAsia100K Scientific Chairman Stephan Schuster

**DOI:** 10.1017/S001667231800006X

**Published:** 2019-03-26

**Authors:** Ian McGonigle, Stephan C. Schuster

**Affiliations:** 1 School of Social Sciences, Nanyang Technological University, Singapore; 2 School of Biological Sciences, Nanyang Technological University, Singapore; 3 Singapore Centre for Environmental Life Sciences Engineering, Nanyang Technological University, Singapore

**Keywords:** biobanks, diversity, ethnicity, Human Genome Project, next-generation sequencing

## Abstract

GenomeAsia100K is a human genome project based at Nanyang Technological University in Singapore that aims to sequence one hundred thousand Asian genomes in an effort that addresses an ethnic bias towards Western populations in previous genomic research. GenomeAsia100K consists of a team of bioinformaticians, statisticians and population geneticists, and was initiated by the Nanyang Technological University in collaboration with industrial partners MedGenome (an Indian R&D company specializing in genomic data), the California Biotech company Genentech, and Macrogen, a genome sequencing company from Korea. The GenomeAsia100K project is amongst the most ambitious precision medicine projects to date but it is not clear how the project will challenge or reshape understandings of ethnic and racial differences in Asian populations. Ian McGonigle, a scientist and cultural anthropologist, sat down with geneticist Stephan C. Schuster, the scientific chairman of GenomeAsia100K, to discuss the project and the implications of genomics for social identity in the 21st century.

“Let's Map the Gap in Genomic Data,” reads the website of GenomeAsia100K, an initiative spearheaded by Professor Stephan C. Schuster at Nanyang Technological University in Singapore in collaboration with Mahesh Pratapneni, Managing Director of India-based investment management firm Emerge Ventures. GenomeAsia100K consists of a team of bioinformaticians and population geneticists and was initiated by Nanyang Technological University (NTU) in collaboration with industrial partners MedGenome – an Indian R&D company specializing in genetic screening – and the California Biotech company Genentech, as well as Macrogen, a genome sequencing company from Korea. The GenomeAsia100K project eventually aims to catalog the genomic data of 100,000 Asians, putting it amongst the most ambitious precision medicine projects to date.

The Singapore-based GenomeAsia100K runs alongside much larger countries that have launched similar efforts. In 2012 the UK Prime Minister David Cameron launched a £300 million, 5-year initiative to sequence 100,000 genomes from UK National Health Service (NHS) patients with rare disorders, cancer and infectious diseases (Marx, 2015). In the US, early in 2015 President Barack Obama announced a $215 million plan to couple patients’ physiological and genetic data to improve the ‘precision’ of individual treatment (White House, 2015). China followed this trend in March 2016, launching the ‘China Precision Medicine Initiative,’ with a $9.2 billion investment to set the country as a global force in precision medicine (Perez, 2017).

These projects stand on the shoulders of past efforts to catalogue human genetic variation, such as the Human Genome Diversity Project, which was launched in the 1990s and which was a major step forward in recognizing the importance of ethnic diversity in medical research. With the advent of second-generation genomic sequencing technologies in recent years, such as the Illumina HiSeq platform that GenomeAsia100K utilizes, full genome sequences can now be generated quickly and more affordably for the purposes of population genetics analysis.

In Singapore, the first stage of work in the GenomeAsia100K project is to sequence 10,000 Asian genomes for ethnic stratification. This initial step will be followed by the sequencing of an additional 90,000 genomes, which will be analysed in relation to medical and phenotype data, examining the role of specific variants in health and disease. This huge level of genomic data generation is facilitated not only by advances over the past decade in genetic sequencing technologies that have sped up the rate of sequencing while rapidly lowering the cost, but also by enhancements in computational power and data storage technologies. Together these technological developments have major social consequences.

Such large-scale genome projects, biobanks and identity-based genetic research raise serious social concerns, including issues in biomedical ethics regarding the legal protection of personal data (Gurwitz, 2015; McGonigle & Shomron, 2016), regarding the definition of the nature of the ‘individual’ participant (McGonigle, 2016), as well as the biopolitics of ethnic inclusion (McGonigle, 2015; McGonigle & Herman, 2015) in this era of the ‘molecularization of identity’ (McGonigle & Benjamin, 2016). Furthermore, the Chinese development aside, so far Asians have been a relatively understudied population when it comes to genetic diversity. In this regard, GenomeAsia100K aims to make a difference.

Although Asians comprise more than 40% of the world's population, these populations “are significantly underrepresented in current genomic studies and reference genome databases.”^1^ But, Asian populations possess genetic diversity that may be “a valuable source of clinical insights to enable cures for all of mankind in rare and inherited diseases, as well as complex diseases such as cancer, diabetes and neurological disorders.”^2^

Authors Assistant Professor Ian McGonigle (IM) and GenomeAsia's Scientific Chairman Professor Stephan C. Schuster (SCS) sat down to discuss the goals of the GenomeAsia100K project, to consider some of the social and cultural challenges it faces in the region, and to talk about how GenomeAsia100K will integrate into the global precision medicine workflow.

## Conversation

**IM: When did you come to Singapore?**

SCS: I came to Singapore in 2011 but starting in 2009 some colleagues and I applied for a large research grant, which we won and used to establish the Singapore Centre for Environmental Life Sciences Engineering (SCELSE), where GenomeAsia100K is based.

**IM: What are your impressions of NTU and the trajectory it is on?**

SCS: There probably are few other places like NTU. This has to do with the recent history of the previous University President, Bertil Andersson, who brought NTU to the top of the global University rankings from outside the top 200 a decade ago.^3^ I think this is quite remarkable given the short period of time. I think that has to do with the way the concept of excellence was very diligently pursued.

**IM: Tell me a little bit about the GenomeAsia100K project and how you came to it.**

SCS: I started a postdoc in a lab in Caltech, not working on genomics. At the lab the technology that enabled the Human Genome Project was developed, that is bacterial artificial clones. Many of us from that lab later moved on to build careers in genomics. For me, that was sequencing bacterial genomes, early on, and from 2009 to 2013 I took a position at Penn State University where I jumped to be the first to sequence ancient DNA using a new sequencing technology. People were initially very disappointed by the error rate of the reads of the new technology but I felt it was a perfect match for damaged ancient DNA. This was the big breakthrough and then it was a turbulent decade during which we demonstrated how this technology can revolutionize biology.

We then sequenced human genome number nine and ten that were published, and they were the South African bushman, the *Khoisan*, and Archbishop Desmond Tutu (Schuster *et al.*, 2010). The idea for this work – that became the *Nature* paper that published this in 2010 – was to include underrepresented minorities in the public databases by generating the alleles for these South Africans that had been excluded in pharmaceutical research. I have to say that this approach really works and today I receive emails from people conducting medical studies where they ask for more people with this kind of variant, because as we have shown in this paper, they are by far the most divergent living humans, but not because of ancient reasons, but because they separated from the rest of humanity 150,000 years ago and then adapted to a very unique lifestyle. So, they are as modern as we are but they have adapted to life in the desert as a hunter–gathering population.

So, this entire idea of using human genomics for societal problems interested me very early on and pretty much now having been in Asia since 2011, it became very clear for almost half of humanity, with Asians being about only 6% of the alleles in the databases. We wanted to generate the genetic map of Asia and this information could be used to specifically separate disease alleles from ethnic alleles. One example of this is in Singapore, if you look at the genomes, about 30% of genomes appear to have thalassemia, but just from the allele frequencies, it is clear that this is nonsense. This underlines that what is based on European alleles as diseases is misrecognized as disease in other ethnic groups. These European alleles are not only European alleles but Northern European alleles, which is also a major part of the immigrants that went to North America. So, this is the source of one of the major biases in the global databases. Over the past 15 years, we have become painfully aware of how these database biases are seriously impacting findings and future research directions. The only way to do this is to go and cover the world and we felt that Asia is wealthy enough to pay for that effort. All of GenomeAsia100K was done by private funding and hosted at NTU, but those funds are not being used for generating proprietary information and patenting but it has been made absolutely clear that this information is for public use. This GenomeAsia100K is pretty much built on the project that we did with Africa.

**IM: 100,000 samples. Why is it so large and is it large enough?**

SCS: The simplest answer is we don't know. Nobody knows as we speak what is the right number to sequence. But it is clear that this is a number that is feasible and we want to use 10%, 10,000 genomes, to generate the map, and the 90,000 to analyse medical cohorts to identify specifically Asian related diseases. As genome sequencing will grow cheaper, this number is likely to go up.

**IM: How do you catalog the different ethnic samples? Is it through national identity, is it racial, is it linguistic? How do you sort out the samples into their reference groups?**

SCS: I would say it is all of this but I would say that nationality is a poor identifier; even language can be a very poor identifier. It is an easy to use proxy but most of all at this phase that we are in we are seeking connections that have been generated by anthropologists. We then do a first pass sequencing, that involves say up to ten individuals of each respective ethnic group, and if we go north of Asia then we soon find out what the average distance between the groups is. So, as we go forward, we generate these rubrics. We have a manuscript in review at the moment where you can see this initial approach.

**IM: In multi-racial populations, there can be multiple components in the population but it is not always politically favourable to challenge notions of indigenous belonging in the nation. How do you think GenomeAsia100K may challenge notions of belonging?**

SCS: I think it strongly will. But at the same time, this is a rapidly emerging field. I would say there are two directions you can take. You look at fossils, historic samples, and depending on culture that is possible or not. So, where bodies were traditionally burnt, you are out of luck. The other one is getting a more and more clear understanding of how rapidly ethnicities change. In Europe, it has become more and more clear that the people who used to live in Europe 4000 years ago are not around anymore. As we are witnessing in daily news, there are streams of migration all through history and I think it is becoming very clear now that these concepts that we are all sons of Genghis Khan or some person in history, whether they existed or not, these ideas will become obsolete.

**IM: What are the challenges of the project? Sometimes there are technical challenges with the sequencing or data storage. Political challenges, in terms of access or ethical approval. Or cultural challenges where there are different levels of trust with the medical community.**

SCS: I would first say that all of what you mention are challenges. We have done quite well to overcome political challenges by just being persistent and very persuasive. And at the same time, our secret shield is that it is absolutely clear that we are using this for good and there are no private interests in the project. I think it is very important that you have absolutely intact ethics and at the same time, the next step is very important that you have ethical clearing for your work. Technical challenges, we have largely overcome. Sequencing is not the bottleneck. It is more getting the institutional approval and funds coming through at the right time.

**IM: What about the politics of inclusion? You mentioned that certain groups have been less well covered in genome sequencing. In this GenomeAsia100K project are there certain nations you are having difficulty getting proportionately included?**

SCS: Of course, absolutely. This is the biggest challenge that we have and this is why we have formed this international consortium and this is why I believe Singapore was the only choice to take the lead on the project, because, put it this way, we are the Switzerland of Asia, and this means banking and banking means keeping things confidential. People need to trust in an intact legal system and in authorities establishing a system where there is confidence and accountability. I think Singapore is also a good place with a proven record of balancing the interests of a multiethnic society.

**IM: This is not a Singaporean national genome project, this is much more about wider Asia.**

SCS: It is absolutely clear from the government side that they did not want to be involved, and we also made clear when we started that we would not use Singaporean samples to start but we would use all the samples that are available in Asia. And it was clear that this is a private initiative and NTU is the only institution that is publicly funded and is a contributor. All the other partners are private entities.

**IM: How does this differ from other genome projects?**

SCS: There are currently mostly three other large genome projects going forward. There is what has spun out of the precision medicine initiative from Obama. It is also very impressive how fast the Genomics England project has progressed over the last six months. They are in a really high gear. I would say ours is the third. And there is the project in Qatar but I am not aware of the latest numbers.

**IM: What about the rest of Asia? India, for example, is very advanced scientifically. Are they doing anything comparable?**

SCS: No, they are part of what we do. They are the largest portion of the sample sequences as we speak in GenomeAsia100K. You will see that in the first publication. By the way, also, many people involved here have sequenced the very first genome in India. This is a company called MedGenome. And they have been partnering with the company Genentech in California, which is a Roche subsidiary. And there is a long record of Roche contributing without taking information private. Now it is Genentech that is putting in substantial effort. They already have shown what kind of benefit can be gained by looking into these populations. For example, India is particularly interesting relative to the Chinese population, as they started from a very small founder population and in record time have ballooned to 1.3 billion and this has resulted in disease allele frequency that is larger than what you would find in the Western population. As a result, the developing world has become finally of interest to global medical research because of the sheer number of rare cases that would be impossible to sample a cohort in the entirety of the US, with 300 million people.

**IM: What has been learned from other genome projects that you have incorporated?**

SCS: I think in principle the technologies and pipelines are so generic that they are easily being shared among the three projects. For example, if you take the US, the Genomics England project, and ours, we would have interfaces that we could instantly share our data because we have absolutely identical ideas about sequence quality, sequencing platforms, sequencing depth, even the software for analysis, they are all identical. It is just a political will that in the end, somebody will say let's make a genome UN and we could easily cover the majority of the planet.

**IM: Who can benefit from the data?**

SCS: I think the human species will be the first species that will have the vast majority of their alleles characterized, leading to personalized therapies.

**IM: How will the public database feed into the global flow to precision medicine.**

SCS: How can precision medicine be precise, if you don't precisely know who you are? Ethnicity will have to become a part of precision medicine.

**IM: You see this database as a necessary upstream precondition for precision medicine?**

SCS: This is where we believe we are ahead of the two other projects. For us, every allele has an ethnic identifier. In a world that is increasingly becoming mixed, many people don't know the genetic mosaic they have. I think we have so many cases already and examples, and we make the case in our first paper that due to what we call global ancestry difference that we paint the chromosomes of each sequence according to ethnicity and this allows you to say that the variant is coming from this background or another background. And one of the very first cases when we found this was with Archbishop Tutu. When we started the project in South Africa he had no idea that he had a relatedness to the bushmen and when he found out he literally cried and he told us the story of how he was denied a South African passport when he was supposed to travel to the White House after he was released from prison and received the Nobel Peace Prize. And he said to us “how can they dare to deny me this passport if I am a son of the earth.”

At the same time, he also said that he now understands why he is so short of stature, despite his Bantu heritage that makes up the majority of his inheritance.

**IM: What about the running of the project? How are the samples collected? Where are they sequenced?**

SCS: Many of the people working on this are young and aspiring scientists, Assistant Professors, postdocs, here at NTU but also at different American and European Universities. The big entry that opened up for us to do this project is the very well kept Horei collection in Japan, a collection of blood and DNA samples. One of my colleagues here, Assistant Professor Hie Lim Kim, who is Korean and was trained in Japan. Over a long process, we convinced the owners of the collection that the samples should not be lost and that the only way to store the samples securely is digitally.

**IM: This is interesting because with multi-racial nations you get a lot of mixing and changing over time. In the Israeli biobank that I studied previously, for example, the biobank has become a kind of biological archive for the first generations of Israelis while in present generations ethnic lines are blurring as the populations mix. So even collections that are not sequenced hold a lot of potential data about the origins of populations and the genetic diversity of ethnic groups.**

SCS: The Bushmen (Khoisan people), and this is the big fight that people would never want to accept, the fact that there are still unmixed bushmen alive.

**IM: It doesn't sound politically correct because it could be used for racist purposes.**

SCS: And when we started this project there were people in the UK that wanted to participate and their institutions prevented them because they believed that we were building the molecular foundation for racism. This is why it was wonderful to have a Peace Prize laureate involved in the project because he was the one who said: “do you really believe I am working with racists?”

The way Africa was colonized by the Europeans has overlooked how well Southern Africans defended the territory, and it wasn't until 1909 that the native indigenous population in today's Namibia met Europeans for the first time at the place where we sampled. The people that we sequenced, aged 80 plus, they were born during the first contact or shortly after.

**IM: So this is very timely, there is a sort of urgency.**

SCS: There is absolute urgency to this. That is exactly the word.

**IM: In cultural anthropology, this is what they called salvage anthropology in the 20th century.**

SCS: I love that term.

**IM: What will be the next step on the project? It will be a public database but what would be done next?**

SCS: I think what needs to be done next is to make all these very complex collections with metadata and this is where mostly statisticians will take their turn and I believe there is a huge opening opportunity for AI projects. Where all the whole-genome association studies have fallen short, when we can build these numbers for these genomes there is a much bigger chance to make more interesting connections, initially as associations, but already we see cases where they found a gene that they found in Parkinson's, but 50% of autism cases had the gene contributing. The same with alleles that associate with schizophrenia, we can see a pattern emerging with towers standing out of the noise that we couldn't see in genome-wide association studies. And the problem with genome-wide association studies is that you only work from the European alleles – this 500,000 that are on the Illumina arrays – and if the variant is not there you miss it. You need all the variants that are in the pool.

There is a paper that Hie Lim Kim and myself published called ‘the poor-man's 1000 Genome Project’ (Kim & Schuster, 2013) in which we work with already sequenced mitochondrial genomes in the database. And what we show is that the degree of genetic variability building up humans so fast is being outbred. We are probably the most outbred mammalian species on the planet. The rate is increasing so fast and this is rapidly impeding our ability to identify diseases. We use this to show how we would have identified mitochondrial genetic disease with what is in the databases. And we show that the noise is so high it is very, very difficult.

Going back to what we talked about earlier, is there anybody who knows how many people we need to sequence to have a sound statistical proof that a variant is linked to this or that observation? And the answer is no.

**IM: What is the timeline for progress?**

SCS: Three to four years. It is not about how fast the sequencing proceeds but how fast the data can be computed.

**IM: What is the limit on computing the data?**

SCS: I would say CPU power but as we move from Sanger to next-generation sequencing we are in the midst of moving from CPU to GPU and this is super interesting because of the sheer power in petaflops that you can get out of these GPU systems is impressive but at the same time these systems are not necessarily being built for genomic analysis where always I/O and memory size are limiting factors.

**IM: How much storage does the project require?**

SCS: For the data storage it is about 25 petabytes but the most important point is that nobody has analysed 100,000 genomes. The variants are being identified in a process that is closed to the public. So, the precision of calling a variant vastly increases if you do not call variants on a single genome but on a group of genomes. So, if you would ask me the question can you conduct variant group calling on 100,000 genomes then I am absolutely certain you currently cannot.

**IM: So am I right to say that if you have say 1000 genomes you do a principal component analysis and come up with different reference populations and the variants become significant within their reference populations?**

SCS: No, this is already based on the variants that you call. So, the step is one step before, where you say which genomes do I pair for the process of group calling.

**IM: That means that these are the constraints, the identities, that you bring to the reading.**

SCS: If you take 950 Europeans and you throw in 50 bushmen then all the variants will be sorted out and you will overlook them. This is why you need to sequence 1000 Bushmen and call out the variants of the 1000 bushmen to make clear that these are ethnic variants from the bushmen, including their rare variants. The way you group your samples vastly determines which alleles are being detected and which are not.

**IM: So, all of the steps of analysis hinge on an initial creation of a reference population, an attribution of some kind of identity.**

SCS: And you need to do this in a very coordinated way. For example, our rule of thumb is we want 25 individuals from an ethnicity, because if it is less than that you can throw in a Neanderthal and you would never know.

**IM: There is an issue of attributing identities that are historically constituted norms. Well, this is where I am very interested in how identities, like national identities, are negotiated in the analysis.**

SCS: This is why you are walking into a minefield. My favorite example always is the Basques. If you look in the literature, there are two kinds of genetic research on Basques, one that has been conducted by Spanish and one conducted by non-Spanish. The results you will find are diametrically opposing. This is why I think anthropological research based on genomes is so interesting, as it makes a case for the UN eventually making political decisions on genetic findings.

**IM: But the genetic findings are downstream of the politics of identity, as you said.**

SCS: This is why you need to let the data speak and you need to be very careful about the process because you can skew the data.

**IM: But there is always a choice how you define the groups.**

SCS: Yes, this is the most important point. How do you identify the groups? For example, I think Bushmen, *Khoisan*, should be granted special rights in Africa, across nations.

**IM: You can inform a lot on historical migrations.**

SCS: Absolutely, and this is what is so interesting. The ancient DNA technologies, that we started in 2005, when we sequenced the mammoth, this is now vastly used for ancient human genomes. It is widely used now for sequencing graves around the world to show for example how herding people are different from hunter-gathering people. And the most interesting part of human history was this transition from hunter–gathering into agriculture – which usually is referred to around 8000 years – but the contact between these two groups in Europe is 4000 years. But in Asia, it could be as little as 1000 years. If you go to Laos or Cambodia you can find hunter–gathering populations to this day. Or you can see the effects of agriculture are very late.

**IM: Regarding the historical implications of GenomeAsia100K, it seems wiser to emphasize the deeper human history and less the modern political history and the contributions to identities.**

SCS: But people always hate this message. This is why I go back to saying this is a minefield. Do you really want to say the Asians had agriculture later than the Arabic cultures? The Indians are fighting the world over who invented zero in mathematics and the Arabic cultures having the monopoly over being the most advanced in mathematics.

**IM: I am very sympathetic to your problems as this is precisely the topic of my research. Anyhow, I should move on to medical issues. Are there going to be clinical records incorporated with the data?**

SCS: Yes, we have a code of conduct and it is determined and regulated. In principle, the stakeholders also collect medical data. Our Indian partners, for example, have trained interviewers in order to collect metadata and not rely on pre-existing medical records.

**IM: How will you get the medical data if you don't have the pre-existing clinical history at the time?**

SCS: This is for the 10% that is for ethnic stratification. For the remaining 90% that are medical cohorts, the medical history will be collected.

**IM: Is there an implication that this work could challenge national identity in Singapore?**

SCS: I think the perception of ethnicity will change globally and this will also impact Singaporeans as well, but I believe that Singapore, striving to be one of the first smart cities, will be one step ahead of this.

**IM: Last question, you mentioned AI briefly, and there is a lot of hype about AI. Is this something you can see helping the project?**

SCS: The word ‘hype’ is what people used to describe next-generation sequencing and man did it transform the world! And yes, there is hype about AI and man how will it transform not only the world, but also genomic research.

**IM: You are very optimistic.**

SCS: We are doing it as we speak. We have a very nice collaboration already with the Computer Science Department at the University of Milan, Bicocca. And it is absolutely clear if you go to conferences, for example, a very interesting one called BioData, the last one was in San Francisco. The head of AI from Google spoke and there can be absolutely no question with the kind of resources that are already being thrown at this that there will be major developments using AI for genomic research.

## Conclusion

Asia has thus far been relatively neglected in the study of human genetic diversity but GenomeAsia100K is well underway to address that shortcoming. GenomeAsia100K not only seeks to map the genetic structure of Asian populations and shed light on the origins of Asian populations but crucially, intends to accelerate “Asian population specific medical advances and precision medicine.”^4^ This type of research that focuses on the distribution of genetic variants and diseases amongst ethnic populations is a growing trend.^5^ In some countries, high rates of endogamy and inheritable disease have created an urgent need for functional genomics research on ethnic populations. For example, *Science* magazine's recent cover feature, “Family ties: Saudi Arabia strives to prevent genetic disorders,” focused on the clinical application of functional genomics in relation to inheritable disorders in Saudi Arabia (Kaiser, 2016). Similarly, its neighbour state Qatar has a genome project that aims to help eliminate genetic disease in the ethnic Qatari population (Fakhro *et al.*, 2016).^6^ Unlike some genomic research projects around the world, however, the GenomeAsia100K project commits to making the genomic data generated public and freely available. The ethos of the effort is in the global humanistic spirit of inclusion and just distribution of the benefits of the latest medical research.

The GenomeAsia100K project also emerges at a timely moment of a growing political awareness – and indeed politicization – of representation and access to medicine and medical research (see, for example, Benjamin, 2013 and Epstein, 2007). Recently, in the US the research program ‘All of Us’ was established to help better include populations that have been underrepresented in biological research. ‘All of Us’ aims “to create a research database that reflects the diversity of the US to fill this gap.”^7^ Dr Stephanie Devaney, Research Program Deputy Director at ‘All of Us’, states “[a]chieving a demographically, geographically and medically diverse participant community is a top priority for us… a diverse participant community will fill gaps in our scientific knowledge and give everyone the chance to benefit from biomedical research.”

The politics of inclusion in Singapore, however, are somewhat distinct to those of the settler colony of the US or indeed to the above-mentioned Gulf monarchies. In the postcolonial context of Singapore and Southeast Asia, rather, when the GenomeAsia100K project launched in 2016, it was reported in the local Singaporean Straits Times newspaper as a database whose “aim is to reduce bias of medicine towards Westerners” (Lin, 2016). The GenomeAsia100K project thus represents a pivot in the focus of global science towards Asia and Asian populations and stands in to fill a regional void in global biomedical research. The project also represents a major step towards the global genomic cataloging of ethnic identities and perhaps, as Stephan Schuster suggests, takes us one step closer towards a UN global genome.

## Footnotes

1 GenomeAsia100K website, accessed 30 July 2018, http://www.genomeasia100k.com/

2 GenomeAsia100K website, accessed 30 July 2018, http://www.genomeasia100k.com/

3 Nanyang Technological University (NTU) placed 11th in the 2018 QS World University Rankings and received a five-star rating, see: https://www.topuniversities.com/university-rankings/world-university-rankings/2018

4 GenomeAsia100K website, accessed 30 July 2018, http://www.genomeasia100k.com/

5 GenomeAsia100K website, accessed 30 July 2018, http://www.genomeasia100k.com/ citing, Rotimi & Jorde (2010).

6 See: Qatar Genome, https://qatargenome.org.qa/

7 Precision Medicine World Conference 2018, accessed online 30 July 2018, https://www.pmwcintl.com/all-of-us-research-program-aims-to-create-diverse-research-data-resource/

## References

[ref1] Benjamin R (2013). People's science: bodies and rights on the stem cell frontier. Palo Alto: Stanford University Press.

[ref2] Epstein S (2007). Inclusion: the politics of difference in medical research. Chicago: University of Chicago Press.

[ref3] Fakhro KA, Staudt MR, Ramstetter MD, Robay A, Malek JA, Badii R, Al-Marri AA, Abi Khalil C, Al-Shakaki A, Chidiac O, Stadler D, Zirie M, Jayyousi A, Salit J, Mezey JG, Crystal RG and Rodriguez-Flores JL (2016). The Qatar genome: a population-specific tool for precision medicine in the Middle East. Human Genome Variation 3, 16016. 27408750 10.1038/hgv.2016.16 PMC4927697

[ref4] Gurwitz D (2015). Genetic privacy: trust is not enough. Science 347(6225), 957–958. 10.1126/science.347.6225.957-b 25722403

[ref5] Kaiser K (2016). When DNA and culture clash: Saudi Arabia is making a big push into human genomics, hoping to prevent inheritable diseases. Science 354(6317), 1217–1221. 27940828 10.1126/science.354.6317.1217

[ref6] Kim HL and Schuster SC (2013). Poor man's 1000 Genome Project: recent human population expansion confounds the detection of disease alleles in 7,098 complete mitochondrial genomes. Frontiers in Genetics 4, 13. 23450075 10.3389/fgene.2013.00013 PMC3584485

[ref7] Lin Y (2016). Sequencing 100,000 Asian genomes for tailor-made healthcare. *The Straits Times* 8 April 2016, p. B14.

[ref9] Marx V (2015). The DNA of a nation. Nature 524(7566), 503–505. 26310768 10.1038/524503a

[ref10] McGonigle IV and Benjamin R (2016). The molecularization of identity: science and subjectivity in the 21st century. Genetics Research 98, e12. 27376979 10.1017/S0016672316000094 PMC6865137

[ref11] McGonigle IV (2016). The collective nature of personalized medicine. Genetics Research 98, e3. 26792757 10.1017/S0016672315000270 PMC6865159

[ref12] McGonigle IV and Shomron N (2016). Privacy, anonymity, and subjectivity, in genomic research. Genetics Research 98, e2. 26763729 10.1017/S0016672315000221 PMC6865168

[ref13] McGonigle IV (2015). ‘Jewish genetics’ and the ‘nature’ of Israeli citizenship. Transversal: Journal for Jewish Studies 13(2), 90–102.

[ref14] McGonigle IV and Herman L (2015). Genetic citizenship: DNA testing and the Israeli law of return. The Journal of Law and the Biosciences 2(2), 469–478. 27774208 10.1093/jlb/lsv027 PMC5034383

[ref15] Perez B (2017). China's ‘precision medicine’ initiative gets lift from latest genomics company funding. Retrieved from http://www.scmp.com/tech/china-tech/article/2092362/chinas-precision-medicine-initiative-gets-lift-latest-genomics

[ref16] Rotimi CN and Jorde LB (2010). Ancestry and disease in the age of genomic medicine. New England Journal of Medicine 363(16), 1551–1558. 20942671 10.1056/NEJMra0911564

[ref17] Schuster SC, Miller W, Ratan A, Tomsho LP, Giardine B, Kasson LR, Harris RS, Petersen DC, Zhao F, Qi J, Alkan C, Kidd JM, Sun Y, Drautz DI, Bouffard P, Muzny DM, Reid JG, Nazareth LV, Wang Q, Burhans R, Riemer C, Wittekindt NE, Moorjani P, Tindall EA, Danko CG, Teo WS, Buboltz AM, Zhang Z, Ma Q, Oosthuysen A, Steenkamp AW, Oostuisen H, Venter P, Gajewski J, Zhang Y, Pugh BF, Makova KD, Nekrutenko A, Mardis ER, Patterson N, Pringle TH, Chiaromonte F, Mullikin JC, Eichler EE, Hardison RC, Gibbs RA, Harkins TT and Hayes VM (2010) Complete *Khoisan* and Bantu genomes from southern Africa. Nature 463(7283), 943–947. 20164927 10.1038/nature08795 PMC3890430

[ref18] White House (2015). Factsheet: President Obama's Precision Medicine Initiative. Press release, 30 January 2015.

