# Global Science Meets Ethnic Diversity: Ian McGonigle interviews GenomeAsia100k Scientific Chairman Stephan Schuster–ADDENDUM

**DOI:** 10.1017/S0016672319000053

**Published:** 2019-05-28

**Authors:** Ian McGonigle, Stephan C. Schuster

**Affiliations:** 1 School of Social Sciences, Nanyang Technological University, Singapore; 2 School of Biological Sciences, Nanyang Technological University, Singapore; 3 Singapore Centre for Environmental Life Sciences Engineering, Nanyang Technological University, Singapore

The abstract in the published article by McGonigle et al, (2019) was incomplete and missed acknowledgement of the role the company Macrogen played in the GenomeAsia100k project. The full abstract should read as below.

GenomeAsia100K is a human genome project based at Nanyang Technological University in Singapore that aims to sequence one hundred thousand Asian genomes in an effort that addresses an ethnic bias towards Western populations in previous genomic research. GenomeAsia100K consists of a team of bioinformaticians, statisticians and population geneticists, and was initiated by the Nanyang Technological University in collaboration with industrial partners MedGenome (an Indian R&D company specializing in genomic data), the California Biotech company Genentech, and Macrogen, a genome sequencing company from Korea. The GenomeAsia100K project is amongst the most ambitious precision medicine projects to date but it is not clear how the project will challenge or reshape understandings of ethnic and racial differences in Asian populations. Ian McGonigle, a scientist and cultural anthropologist, sat down with geneticist Stephan C. Schuster, the scientific chairman of GenomeAsia100K, to discuss the project and the implications of genomics for social identity in the 21st century.

The online version of the article has been updated to include the complete abstract.
